# A Comparative Study on the Viability of Normal and Cancerous Cells upon Irradiation with a Steady Beam of THz Rays

**DOI:** 10.3390/life12030376

**Published:** 2022-03-05

**Authors:** Larysa Kovalevska, Olexandr Golenkov, Yelyzaveta Kulahina, Todd Callender, Fedir Sizov, Elena Kashuba

**Affiliations:** 1 R.E. Kavetsky Institute of Experimental Pathology, Oncology and Radiobiology, National Academy of Sciences of Ukraine, 45 Vasylkivska Str., 03022 Kyiv, Ukraine; kreyl@yahoo.com (L.K.); lkulagina.2001.sumy@gmail.com (Y.K.); 2 V. Lashkaryov Institute of Semiconductor Physics, National Academy of Sciences of Ukraine, 41 pr Nauki, 03028 Kyiv, Ukraine; golenkov@isp.kiev.ua (O.G.); sizov@isp.kiev.ua (F.S.); 3 The Cotswold Group of Companies, Goodman’s Bay Corporate Center, West Bay Street, Nassau P.O. Box CB 12762, Bahamas; todd.callender@cotswoldgroup.net

**Keywords:** terahertz (THz) irradiation, blood cells, cancer cells, apoptosis, cell death

## Abstract

Terahertz (THz) electromagnetic radiation is commonly used in astronomy, security screening, imaging, and biomedicine, among other applications. Such approach has raised the question of the influence of THz irradiation on biological objects, especially the human body. However, the results obtained to date are quite controversial. Therefore, we performed a comparative study on the viability of normal cells and cancer cells upon irradiation with a steady beam of THz rays. We used human peripheral blood mononuclear cells and cancer cell lines. Primary human mononuclear blood cells (monocytes, and B-, and T-cells) showed an increased death rate, determined by cell counting and fluorescence microscopy, upon 0.14 THz irradiation. The effect of THz radiation was different among malignant cells of B- and T-cell origin (Ramos and Jurkat cells) and epithelial cancer cells (MCF7 and LNCaP). This was demonstrated by cell counting and by the alamarBlue assay. In conclusion, THz radiation can result in the death of human primary and malignant cells. However, the mechanism of this phenomenon is largely unknown. Hence, more work should be done to shed some light on the mechanism of action of THz irradiation in living organisms to enhance technologic developments.

## 1. Introduction

Modern technologies based on the use of infrared (IR) radiation and, particularly, terahertz (THz) electromagnetic waves are widely applied in biomedical sciences and continue to undergo a rapid development. It is accepted that, considering the wavelength, the IR region is situated between 0.75 and 30 µm, corresponding to the range of radiation frequencies (ν) of 400–10 THz, while the THz region is located in the range of 30 µm–3 mm, with ν = 0.1–10 THz [1]. Hence, the THz region corresponds to a transition area between far IR and microwaves (λ > 3 mm). 

THz radiation has drawn much attention due to its applications in astronomy, security screening, imaging, biomedicine, among others. Such broad application has raised the question of the influence of THz irradiation on biological objects, especially on the hu-man body. This question has been addressed using various model systems both, in vitro and in vivo. However, the results remain controversial.

THz radiation is nonionizing, and the rays can penetrate paper, plastic, and up to 300 µm in the human skin [1]. Due to the influence of THz radiation on the vibration of atomic groups in macromolecules and on hydrogen bonds, heating is expected to occur upon irradiation [2,3]. With low photon energies, THz radiation is considered safe for humans because it is nonionizing (e.g., 1 THz photon energy <0.004 eV). However, exposure to relatively large energy levels can be harmful because of the thermal effects. Even so, the maximal increase in temperature observed in primary human skin fibroblasts treated with THz pulsed radiation (1.5 THz, power P = 1.5 mW, intensity I = 0.83 W/cm^2^, t = 1000 s) was only 0.7 °C [3]. Upon irradiation (2.7 THz, I = 260 mW/cm^2^, t = 30 min) of the skin of a living mouse, no heating of the tissue was detected [4].

It is noteworthy that no changes in cell morphology, viability, or genetic make-up were found after irradiation with THz of other cell types, e.g., human skin cells (0.380 and 2.520 THz, I = 0.03–0.9 mW/cm^2^, for 2 and 8 h) [5], fetal fibroblasts (0.1–0.15 THz) [6], human embryonic stem cells (hESCs) (2.3 THz, I = 140 mW/cm^2^ for 1 h) [7], transformed corneal epithelial cells (HCE-T), retinal pigment epithelial cells (ARPE-19), and hESCs (0.5 THz) [8].

Others observed induced DNA damage in irradiated artificial skin (0.1–2 THz, I = 57 mW/cm^2^, t = 2 min) [9], phosphorylation of H2A histone family member X in irradiated human primary skin fibroblasts (1.5–3 THz, I—0.84 mW/cm^2^—32 GW/cm^2^, t—10–180 min) [10], genomic instability in human lymphocytes (0.1 THz, I = 0.031 mW/cm^2^, t = 1, 2, and 24 h) [11], aneuploidy in primary adult skin fibroblasts (0.10–0.15 THz, I = 0.40 mW/cm^2^) [12], and disturbances in cell division (inhibition of cytokinesis) in human cervical cancer HeLa cells (0.28–0.46 THz, I = 6–125 mW/cm^2^, t = 1 h) [13].

According to the standards for safety levels with respect to human exposure to radiofrequency electromagnetic fields, developed by the Institute of Electrical and Electronics Engineers (IEEE), THz and microwave radiation is considered safe when 1 ≤ I ≤ 10 mW/cm^2^ [14]. Therefore, considering the upper limit of THz radiation exposure (radiation frequency 0.14 THz) with I = 10 mW/cm^2^, we aimed to perform a comparative study to qualitatively evaluate the viability of normal and cancerous cells upon irradiation with a steady beam of THz rays.

## 2. Materials and Methods

### 2.1. Irradiation Characteristics

**Figure 1:**
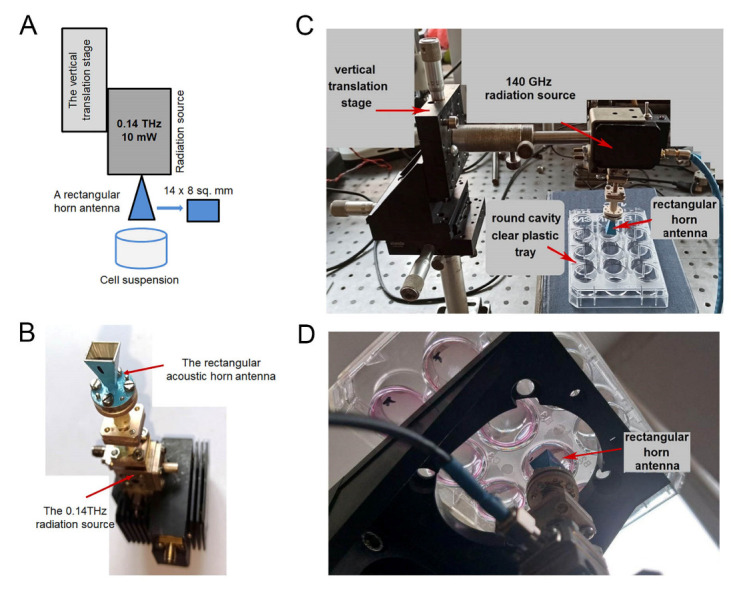
Schema of the experimental system. (**A**) schematic view; (**B**) photograph of the radiation source and the horn (upside down); (**C**) photo of the experimental system, including the 12-well plastic plate for the suspended cells, when the irradiation was from above; (**D**) photo of the experimental system, when cells were irradiated from the bottom.

An experimental system was constructed, consisting of a radiation source composed of a rectangular horn antenna, a vertical translation stage, a well in a polystyrene plate, and a timer (Figure 1). The radiation source was based on the IMPATT (impact ionization avalanche transit-time) diode principle. It could work as a continuous wave or as a modulated power source. The output radiation was linearly polarized, with power (P) = 10 mW. The rectangular horn antenna (Figure 1B–D) was joined to the radiation source via a WR-6 waveguide. The radiation P was measured by a THZ12D-3S-VP-D0 THz detector (Gentec Electro-Optics, Quebec-City, QC, Canada). The length and width of the horn were 14 mm and 8 mm, respectively, with an area of S = 112 mm^2^.

The vertical translation stage was used to adjust the distance between the horn antenna and the surface of a cell suspension placed in 12-well polystyrene plates. The volume of the cell suspension was 2 mL in each well (20 mm in diameter) (Figure 1C,D). The distance between the rectangular horn antenna and the surface of the cell suspension (h_0_) was 1 mm. The average radiation intensity (I) near the surface of the cell suspension could be calculated as I = P/(k∙S) = 8.9/*k* mW/cm^2^, where *k* = 1.2 is the coefficient, depending on the distance h_0_. 

Human cells were irradiated with rays at a wavelength of λ = 2.14 mm, i.e., the frequency (ν) was about 140 GHz or 0.14 THz. Two experimental set ups were used, namely, cells were illuminated from above (Figure 1C) and from the bottom of the well (Figure 1D).

The cells were irradiated in the suspension either in phosphate-buffered saline (PBS; 137 mM NaCl, 2.7 mM KCl, 10 mM Na2HPO4, 1.8 mM KH2PO4, pH = 7.4) or in Iscove′s modified Dulbecco′s medium (IMDM) that contained a high glucose concentration (4500 mg/L), sodium pyruvate, additional amino acids, HEPES buffer, selenium, and L-glutamine), supplemented with 5% (*v*/*v*) fetal bovine serum (FBS) and appropriate antibiotics (all from Thermo Fisher Scientific, Waltham, MA, USA).

### 2.2. Isolation of Human Peripheral Blood Mononuclear Cells

Human mononuclear cells (B- and T-lymphocytes, natural killer (NK) cells, and monocytes) were isolated from human buffy coat blood on Lymphoprep (polysaccharide, 5.7% *w*/*v*; sodium diatrizoate, 9.1% *w*/*v*) gradients (Thermo Fisher Scientific). Mononuclear cells were freshly isolated in each round of experiments. After isolation, the cells were suspended in FBS and stored at +4 °C for no more than 2 days to avoid spontaneous apoptosis.

### 2.3. Cancer Cell Cultures

The following human cancer cells were studied: Burkitt lymphoma Ramos cells (Epstein–Barr virus-negative, with homozygous mutated TP53 [15]); T-cell acute lymphoblastic leukemia Jurkat cells; invasive breast carcinoma MCF7 cells (wild-type TP53); prostate cancer cells LNCaP (wild-type p53 [16]).

Cells were grown in a humidified, CO2 (5% vol.) incubator at 37 °C in IMDM, supplemented with 5% (*v*/*v*) FBS and appropriate antibiotics (penicillin (100 IU/mL) and streptomycin (100 µg/mL); Thermo Fisher Scientific).

### 2.4. Cell Count and Metabolic Activity Assay

The cell count was performed in a Burker chamber (Glaswarenfabrik Karl Hecht KG, Sondheim, Germany), using a trypan blue (Sigma-Aldrich, St. Louis, MO, USA) solution (0.4%) to distinguish between viable and apoptotic (necrotic) cells. This dye can penetrate the cell membrane when it is damaged, making cells appear blue, while viable cells remain transparent.

The number of metabolically active cells was determined using an alamarBlue assay (Thermo Fisher Scientific) with negative controls [17]. This method is based on the observation that when the nonfluorescent reagent resazurin enters living cells, it is reduced to resorufin, a fluorescent molecule. Cell metabolic activity (a surrogate of viability) that is proportional to and corresponds to a specific level of resazurin reduction, was calculated based on differences in absorption at 540 nm and 630 nm. Absorption was measured using a Labsytems Multiskan PLUS spectrofluorometer (Thermo Fisher Scientific), according to the manufacturer’s protocol.

### 2.5. Fluorescent Microscopy and Cell Staining

After irradiation, mononuclear cells were fixed in a mixture of methanol and acetone (1:1) and kept at −20 °C. Prior to staining with primary mouse antibodies against CD14, CD3, and CD19 (Thermo Fisher Scientific), the cells were rehydrated in PBS for 30 min. The cells were incubated with primary antibodies for 1 h and with a secondary rabbit anti-mouse FITC-conjugated antibody (Dako, Glostrup, Denmark) for 30 min at room temperature. Hoechst 3334 was used for DNA staining. Images were captured with the use of a fluorescence microscope (DAS microscope Leitz DM RB with a dual mode cooled charged coupled device (CCD) camera C4880, Hamamatsu).

## 3. Results

### 3.1. Mononuclear Cells Are Sensitive to THz Radiation

At first, the experimental system depicted in Figure 1C was used. The suspension of primary cells isolated from peripheral blood, contained a mixture of mononuclear cells, namely, B- and T-lymphocytes, NK cells, and monocytes. The initial concentration of the cells was approximately 400,000 cells per 1 mL, as determined by the count with trypan blue. In each well, 2 mL of cell suspension was used. Bovine serum can be considered a protein–salt aqueous solution. Experiments were performed two times, each time in triplicates (three wells for each cell type). The number of viable cells was determined using the trypan blue solution after irradiation. We did not use the alamarBlue assay for this part of the study because the mononucleated cells were not activated; thus, they would not proliferate in vitro. To monitor changes in the temperature of a water-based suspension, we treated PBS with the 0.14 THz radiation in the same conditions. In 60 min, the change ΔT was ≤0.2 °C (without shielding from the air flow). Hence, such small fluctuations in the temperature could be neglected. Moreover, such changes in the temperature could not influence cell viability.

**Figure 2:**
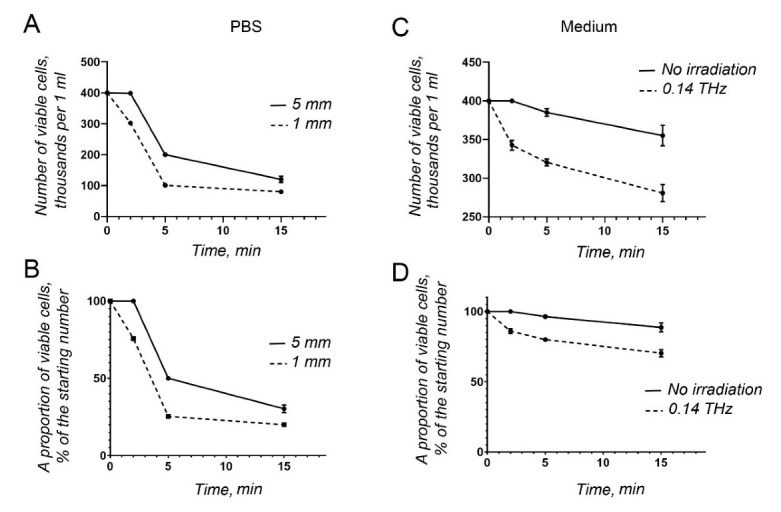
Cell viability upon THz irradiation. (**A**) The absolute number obtained by cell counting, while the horn was placed at different distances from the surface of the cell suspension; (**B**) as in (**A**), calculated as a proportion of viable cells with respect to the original cell concentration. (**C**) absolute number from the cell count, upon irradiation and without irradiation; (**D**) as in (**C**), calculated as a proportion of viable cells with respect to the original cell concentration. Cells depicted in (**A**,**B**) were suspended in PBS, and those in (**C**,**D**) in IMDM supplemented with FBS and antibiotics.

First, the optimal distance between the horn and the surface of the cell suspension was determined (Figure 2A,B). Obviously, the smaller the distance, the better the interaction between cells in suspension and THz irradiation.

When the cells were suspended in PBS, they died quickly (Figure 2A,B). Based on this observation, IMDM was used subsequently. To compare the kinetics of cell death, primary apoptosis-prone mononucleated blood cells were either irradiated (T = 20–22 °C, a pressure of 100–100.7 kPa) or kept in the same conditions without radiation. We could detect a diminishing number of primary mononucleated blood cells even with no radiation (Figure 2C,D). However, even less viable cells could be found following THz irradiation (Figure 2C,D).

**Figure 3:**
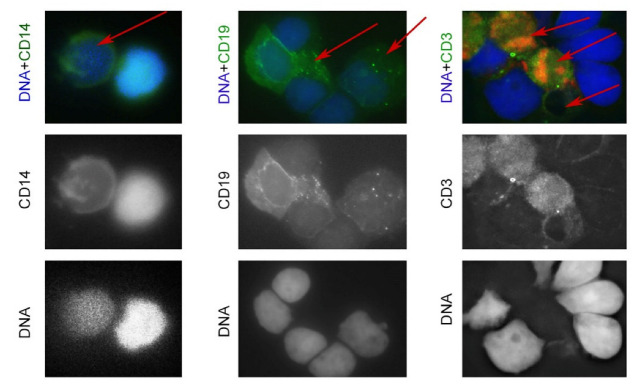
Viability of mononucleated cells upon THz irradiation. Cells were stained with antibodies against CD14 (monocyte marker), CD19 (B-cell marker), and CD3 (T-cell marker).

Hence, the primary fragile human mononuclear blood cells showed an increased death rate upon 0.14 THz irradiation. Next, we examined mononucleated cells stained with anti-CD14, a marker of monocytes (macrophages), CD19, a B-cell marker, and anti-CD3, a T-cell marker. As expected, a heterogeneous cell population was observed, consisting of living cells (double-stained, indicated by the green and blue colors, Figure 3) and dead or dying cells (Figure 3, indicated by red arrows).

The observed enhanced cell death was quite unexpected. Therefore, we next decided to analyze the viability of human cancer cells in similar conditions. To do so, we chose malignant cells of B- and T-cell origin (Ramos and Jurkat cells, respectively) and epithelial cancer cells, representing the most often diagnosed tumors, i.e., breast adenocarcinoma (MCF7) and prostate carcinoma (LNCaP).

### 3.2. Effects of THz Irradiation on Cancer Cells

In the first round of experiments, the set-up was the same as for the mononuclear cells (depicted on Figure 1C). Cell suspensions in IMDM were used, also for epithelial tumor cells. Of note, the chosen cancerous cells are not prone to induced cell death at room temperature for at least a couple of hours. 

**Figure 4:**
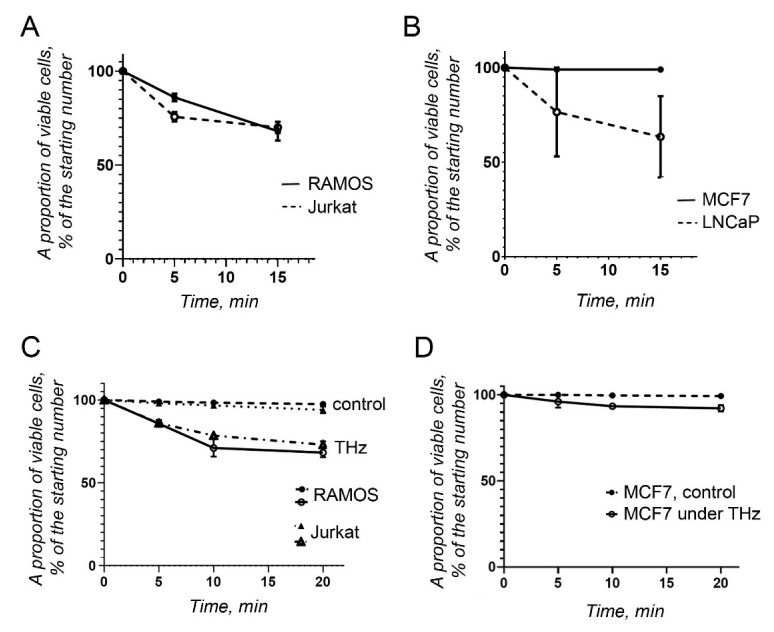
Viability of cancer cells upon THz irradiation. (**A**) Proportion of viable cancer cells, derived from Burkitt’s lymphoma (Ramos) and T-cell acute lymphoblastic leukemia (Jurkat), calculated from the original cell concentration, when cell suspensions were irradiated from the surface; (**B**) as in (**A**), proportion of viable cancer cells, derived from breast adenocarcinoma (MCF7) and prostate cancer (LNCaP); (**C**) as in (**A**), but the cells were irradiated through the bottom of the wells; (**D**) As in (**B**), but MCF7 adherent cells were irradiated through the bottom of the wells.

The cancer cells of lymphocytic origin, Ramos and Jurkat cells, showed a moderate reaction upon irradiation with THz waves. After 15 min of irradiation, both cell lines showed a similar level of viability, corresponding to approximately 75% (Figure 4A). In this sense, they behaved similarly to primary mononuclear blood cells (Figure 2C,D).

A differential response to THz radiation was observed in cancer cells of epithelial origin, MCF7 and LNCaP (Figure 4B). MCF7 cells were quite stable and did not show any sign of cell death or apoptosis. By contrast, LNCaP prostate cancer cells were affected significantly, and after 15 min of irradiation, only 63% of cells, on average, were still alive.

Next, the experimental system depicted on Figure 1D was used. The cells were in the same conditions, as described above; however, the THz irradiation beam was applied from the bottom of the well.

The viability of the irradiated and non-irradiated cells was compared (Figure 4C,D). Importantly, the number of the viable cells in suspension was almost the same for Ramos and Jurkat cell lines (Figure 4A,C). There were slightly less viable adherent MCF7 cells, compared with the first round of experiments (Figure 4B,D). 

**Figure 5:**
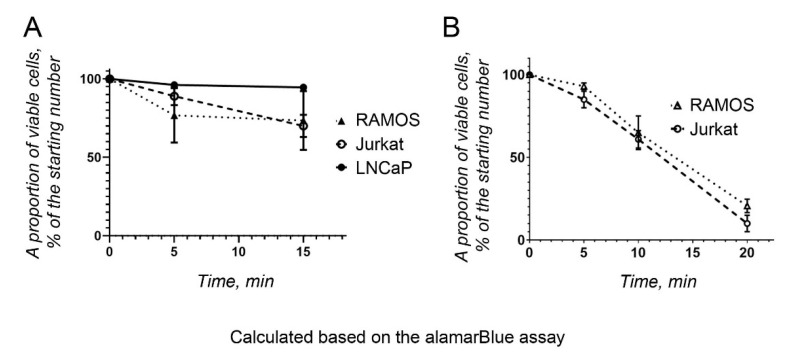
Viability of cancer cells upon THz irradiation. The proportion of metabolically active cancer cells was calculated with respect to the original cell concentration using the alamarBlue assay. (**A**) THz irradiation from the surface of the cells. (**B**) THz irradiation from the bottom of the well.

To monitor the metabolic activity of the cancer cells after irradiation, the alamarBlue assay was performed on the four cell lines described above. Briefly, a resazurin stock solution (440 µM) was added to the cells after irradiation, and the cells were incubated for 4 h. After spectrophotometry, the proportion of reduced resazurin was calculated as a measure of the proportion of metabolically active cells (Figure 5). The test was performed for a short period of time. The test results were compared with those obtained using a resazurin solution in IMDM, supplemented with FBS and antibiotics, in the absence of cells. The nonirradiated cells were also kept as a cell suspension to make the conditions comparable with those of the irradiated cells. MCF7 cells were excluded from the alamarBlue assay because they were resistant to THz irradiation.

Notably, the results obtained with the alamarBlue assay differed for the various cell lines, compared with those obtained with the untreated cells (Figure 5). Thus, the number of viable Ramos and Jurkat cells was similar to that counted earlier using trypan blue (Figure 4A and Figure 5A), when cells were irradiated from the surface (Figure 1C). These cells were quite sensitive to THz radiation. However, LNCaP cells showed the highest metabolic activity after irradiation (Figure 5A), in contrast to the results obtained earlier (Figure 4B). The alamarBlue viability test could yield “false positive” results, because the medium itself can reduce resazurin to a certain degree (17), which should be considered when only one test is used to assess cell viability.

When the cells were irradiated from the bottom of the well (Figure 1D), the number of metabolically active cells decreased significantly, reaching approximately 20% after 20 min of THz irradiation (Figure 5B). 

## 4. Discussion

The question of the influence of THz radiation on living organisms has become intriguing with the increasing volume of related new experimental data. As was mentioned in the Introduction, the experimental data on the effect of THz radiation on living cells are contradictory. On the one hand, no DNA damage was detected by various authors [5,6,7], while others could demonstrate DNA damage [8] and increased aneuploidy [12]. It was reported that THz rays could induce the differentiation of mouse mesenchymal stem cells into adipocytes via the activation of peroxisome proliferator-activated receptor gamma (PPARG) [18]. However, without an at least qualitative lipid staining, e.g., with Oil red O, it is not possible to conclude that “lipid droplet-like structures” are really lipid droplets. Autophagic vacuoles look the same, and it is possible that THz radiation induces autophagy and, eventually, cell death.

No changes in morphology, attachment, proliferation, and differentiation were observed in epithelial cell lines of different origin upon irradiation with 0.1–2.52 THz, as discussed earlier. No significant changes in DNA methylation and gene expression patterns were reported. Thus, heat shock, cytoskeleton, and prosurvival genes and proteins were expressed at roughly the same level prior to and upon THz radiation [6,7,10,19,20,21]. The fold change was 2–2.5 times that observed at the maximum; most of the values showed 0.8–1.5-fold differences. This could easily be explained by a measurement error in the quantitative polymerase chain reaction method used for the control experiments after RNA sequencing or other “omics” methods of comparison.

Importantly, it was shown that the cell membrane became more permeable [22] under THz irradiation (10 min, 0.3–19.5 THz), as demonstrated with the help of fluorescent silica nanospheres (diameter, approximately 23 nm). Similar results indicating altered endocytosis upon the THz irradiation (3.1 THz) of neuronal cells were recently reported [23]. A conclusion was made, based on the changes in the intensity of the signal of a membrane-selective fluorescent dye FM4-64 upon the irradiation. Moreover, it was shown that the THz rays influenced F-actin polymerization both in vitro [24] and in vivo, in living cells [13,25]. Unfortunately, the effect described in viable cells was quite different—the destruction of actin filaments [25] and the induction of F-actin and its polarization [13] was observed. Most likely, similar observations could be interpreted differently.

It was proposed that epigenetic changes might be caused by THz irradiation, such as phosphorylation of the histone H2AX in human primary skin fibroblasts [10]. On the contrary, phosphorylated H2AX (γH2Ax) was not detected in hESCs [7], as mentioned earlier. Noteworthy, the formation of γH2Ax foci and their number do not prove the appearance of double-strand DNA breaks or their role in DNA repair [26].

Importantly, it was found, using a nuclear magnetic resonance technique, that the irradiation of ubiquitin in a H_2_O/D_2_O solution (0.1 THz) can influence the hydrogen bond network surrounding a protein molecule [27]. Moreover, using electron paramagnetic resonance, it was shown that THz irradiation (0.2–1.5 THz) not only influenced the hydrogen bond network of the albumin molecule (in a water solution), but also increased the rate of intra- and inter-molecular interactions [28].

Despite the lack of knowledge about the molecular mechanisms of putative changes in nucleic acids and proteins evoked by THz irradiation, the consequences might be severe for mammalian cells and tissues. Thus, our experiments showed that THz irradiation of human lymphocytes may lead to cell death. It is plausible that THz waves of a certain frequency can influence supramolecular biological assemblies, namely, protein–protein/protein–DNA/protein–RNA complexes, starting an avalanche of molecular events. This question, no doubt, should be further investigated.

## 5. Conclusions

THz radiation can result in the death of human mononuclear blood cells. However, the mechanism of this phenomenon is largely unexplained. More work should be done to shed some light on the mechanisms of action of THz irradiation in living organisms to speed up technological development.

## Data Availability

All experimental data supporting the reported results can be delivered upon the request.
